# Loneliness Across the COVID-19 Pandemic: Risk Factors in Norwegian Young People

**DOI:** 10.32872/cpe.10483

**Published:** 2023-09-29

**Authors:** Mari Hysing, Keith J. Petrie, Allison G. Harvey, Kari-Jussie Lønning, Børge Sivertsen

**Affiliations:** 1 Department of Psychosocial Science, Faculty of Psychology, University of Bergen, Bergen, Norway; 2 Department of Psychological Medicine, University of Auckland, Auckland, New Zealand; 3 Department of Psychology, University of California, Berkeley, CA, USA; 4 Modum Bad Psychiatric Hospital, Vikersund, Norway; 5 The Student Welfare Organization in Oslo and Akershus (SiO), Oslo, Norway; 6 Department of Health Promotion, Norwegian Institute of Public Health, Bergen, Norway; 7 Department of Research & Innovation, Helse-Fonna HF, Haugesund, Norway; Philipps-University of Marburg, Marburg, Germany

**Keywords:** loneliness, social isolation, mental health, young adult, COVID-19

## Abstract

**Background:**

There is evidence of increasing levels of loneliness in Norwegian young people before the COVID-19 pandemic. It is not clear how the COVID-19 pandemic, and the associated necessary restrictions, impacted on these trends.

**Aims:**

To examine how loneliness in young people changed across the pandemic, how loneliness relates to demographic characteristics and how different pandemic restrictions impacted loneliness.

**Method:**

We analyzed data from three waves of a Norwegian national higher education student survey (the SHoT-study). Data was examined from 2018 from a total of 49,836 students, 2021 from 62,212 students, and from 2022 from 53,362 (response rates 31-35%). Loneliness was measured by “The Three-Item Loneliness Scale” (T-ILS).

**Results:**

There was a sharp increase in loneliness from 2018 to 2021, and a reduction in levels of loneliness in 2022, although at increased levels compared to prior to the pandemic. Females consistently report higher levels of loneliness than males, with a larger difference during the peak of the pandemic. There were higher rates of loneliness in geographical regions with higher COVID rates and greater pandemic-related restrictions during 2021. Loneliness was lower among students reporting more days on campus in 2021 and for those with lectures on campus in 2022, both with dose-response associations.

**Conclusions:**

Loneliness is a major public health problem among young adults in higher education. Loneliness increased during the pandemic and has decreased but is still not back to pre-pandemic levels. The results suggest the importance of open campuses and in-person lectures, for increased social connectedness among young people.

## Highlights

- Loneliness increased among university students from 2018 to 2021 and decreased from 2021 to 2022, but was still higher in 2022 than pre-pandemic levels.
- Loneliness was higher in areas with higher restriction levels in 2021.
- Spending time on campus was associated with lower levels of loneliness.
- Online learning was related to higher levels of loneliness.

Loneliness is often described as a perceived deficiency in social relationships and is associated with a number of negative psychological and physical health outcomes (Hawkley & Cacioppo, 2010). There is growing recognition of loneliness as a significant public health issue with negative effects comparable to risk factors such as physical inactivity, obesity and smoking (Holt-Lunstad et al., 2017; Holt-Lunstad et al., 2010) A recent review of the prevalence of loneliness prior to the COVID-19 pandemic indicated heterogenous but at substantial levels of loneliness in many countries (Surkalim et al., 2022).

There is also evidence that loneliness is increasing in young adults. A recent meta-analysis and systematic review of 345 studies of adults aged 18-29 who completed the UCLA Loneliness scale between 1976 and 2019 found loneliness levels increased linearly each year (Buecker et al., 2021). Consistent with this pattern, Hysing and colleagues (2020) highlighted an increase in loneliness among Norwegian fulltime students from 2014 to 2018 with an overall increase in students feeling lonely from 16% to 23%. The study also found males reported the greatest increase in loneliness over time. However, it is not known if this trend continued. Based on pre-pandemic studies, the gender differences in loneliness have been inconsistent. On the one hand, two meta-analyses concluded that males had higher levels of loneliness (Maes et al., 2019; Mahon et al., 2006). On the other hand, a higher level of loneliness has been observed among women, relative to men, among young adults (Wickens et al., 2021). We have previously found that the youngest and oldest students reported the highest levels of loneliness pre-pandemic (Hysing et al., 2020), and the youngest may be at an extra risk of loneliness during the pandemic since they may not have established social networks.

The COVID-19 pandemic in 2020 gave rise to strict social restrictions and government mandated lockdowns in most countries to combat the spread of the virus. In Norway there were both national restrictions, and regional restrictions during the pandemic based on COVID rates (Han et al., 2020). For university students, a range of COVID-19 preventive measures impacted their everyday life from social distancing restrictions in the population at large to closed campuses and restrictions on time on campus and reliance on online teaching (Han et al., 2020). There were both regional differences in restriction level, but also differences in the transition from online to campus-based teaching when the restrictions were lifted. For university students, these restrictions on social activities and reliance on online education may have set the scene for an even further increase in the rate of loneliness. This is confirmed by unprecedented high levels of loneliness reported among young adults during periods of pandemic restrictions (Horigian et al., 2021; Padmanabhanunni & Pretorius, 2021; Sigfridsson & Brandt, 2021). The UK COVID-19 Social Study found young adults were at greater risk of loneliness during the pandemic, compared to pre-pandemic. Also, being a student was an increased risk for loneliness (Bu et al., 2020). Similarly, we have previously found that mental health problems were more prevalent among students in areas with a higher level of restrictions for going onto campus and greater online learning (Sivertsen et al., 2022).

The aim of the present study is to assess changes from prior to the pandemic (2018) to a period of restrictions for students during the pandemic (2021) and after most of the restrictions were lifted (2022). Further, we will assess if loneliness levels differ across key sociodemographic groups. Given the contradictory findings, we do not have a specific hypothesis regarding gender differences in loneliness. However, we hypothesize that younger students will report higher loneliness levels over time. Further, loneliness is expected to be higher in areas with high restriction levels during the pandemic and with more online and off-campus learning.

## Method

### Procedure

The SHoT study (Students' Health and Wellbeing Study) is a large Norwegian survey of students in higher education, conducted by three large student welfare organizations. Five surveys have been completed since 2010. This report is based on the three latest waves, conducted in 2018, 2021 and 2022. The SHoT 2018 and the SHoT 2022 were both conducted between February and April. SHoT 2021 was a briefer version focusing specifically on the COVID19 pandemic. SHoT 2021 was conducted between March and April. All full-time Norwegian students pursuing higher education were invited to participate. For SHoT 2018, SHoT 2021 and SHoT 2022, 162,512, 181,828, and 169,572 students fulfilled the inclusion criteria, of whom 50,054 (response rate: 30.8%), 62,498 (response rate 34.4%) and 59,554 (response rate: 35.1%) students completed the online questionnaires, respectively. In 2018, only students aged 18 to 35 years were included, while the 2021- and 2022-studies also included students older than 35. To enable comparisons across the three time points, the current study included students aged 18 to 35 years, yielding final sample sizes of 49,836 (2018), and 62,212 (2021), and 53,362 (2022). Detailed information of the SHoT study has been described elsewhere (Sivertsen et al., 2019).

#### Data Collection and Pandemic Restrictions

In Norway, the national and regional restrictions triggered by the COVID-19 pandemic changed over time. During the 2021 data collection, there were both national and regional restrictions, and there was mainly online teaching for the students and closed campuses, with some exceptions. For the 2022 data collection, there was still an ongoing pandemic, but the national and regional restrictions had lifted in Norway just before the data collection started. Still, some restrictions were in place and a hybrid of live and online teaching was offered.

#### Statistical Analyses

IBM SPSS Statistics 28 for Windows (SPSS Inc., Chicago, IL) was used for all statistical analyses. Pearson’s chi-squared tests were used to examine changes in the prevalence of loneliness (the three T-ILS items) for male and female students separately. The magnitude of gender differences was examined using Cohen’s *h*, which is measure of distance between two proportions (and interpreted similarly to Cohen’s *d*). Chi-squared tests were also used to examine the association between loneliness and age group, and levels of campus closure (SHoT 2021) and online lectures (SHoT 2022). Geographical differences in loneliness (T-ILS) in the SHoT 2021 were examined by computing Estimated Marginal Means, means adjusting for sociodemographic factors (age, sex, relationship status and ethnicity), and COVID-19 factors (# of tests, positive test, having been in quarantine). There was generally very little missing data on the included variables across all three waves, and the missing values were handled using listwise deletion.

#### Ethics

All procedures involving human subjects/patients were approved by the Regional Committee for Medical and Health Research Ethics in Western Norway (SHoT 2018: no. 2017/1176, SHoT 2021: no. 176205, and SHoT 2022: no. 326437, respectively). Electronic informed consent was obtained after complete description of the study to the participants. Following completion of the surveys, the participants had received detailed information about the findings.

#### Patient and Public Involvement

The planning and design of all three SHoT studies were initiated and governed by the three largest student welfare organizations, which included deciding inclusion and exclusion criteria, and selecting potential research questions and instruments. Students were not involved in the actual collection of data, although recruitment was conducted in close collaboration with all the student welfare organizations in Norway.

### Instruments

#### Demographic and COVID-19-Related Information

In all three SHoT studies, the students provided data on their age, gender, relationship status (single versus married/partner/boyfriend/girlfriend) and the education attained by their parents. Indication of gender had three response options: “woman,” “man” and “other”. Ethnicity was coded as Norwegian if the student or his/her parents were born in Norway, and “other” for all other countries. Based on the geographical location of each educational institution, students were categorized according to Norway’s recent county reform, which now includes 10 counties.

In the SHoT 2021 study, all students were also asked how many days they had physically spent on campus during the last 14 days, due to COVID-19 restrictions. In 2022, respondents were asked how much of the teaching had been online since fall, 2021. They also reported if they had been tested for COVID-19, number of tests, positive test (confirmed by an established test), and whether they had been in quarantine (which typically entails 10 days of staying at home/avoiding social contact).

#### Loneliness

In all three SHoT studies, loneliness was assessed using an abbreviated version of the widely used UCLA Loneliness Scale, the “Three-Item Loneliness Scale (T-ILS)” (Hughes et al., 2004). The T-ILS items (lack of companionship, feeling left out, and isolation) were each rated along a 5-point scale (“never”, “seldom”, “sometimes”, “often”, and “very often”). The T-ILS has displayed satisfactory reliability and both concurrent and discriminant validity (Hughes et al., 2004). More information about loneliness in the SHoT study has been published elsewhere (Hysing et al., 2020). In addition, the SHoT 2022 study also included a single item assessing to what extent the student felt s/he had enough friends at their campus, with the response options “I have many friends”, “I have some friends”, “I have few friends”, and “I have no friends”. The Cronbach’s alphas of the T-ILS were 0.87 (2022), 0.84 (2021), and 0.88 (2018).

## Results

### Sample Characteristics

As detailed in Table 1, female students comprised approximately 2/3 of the participants in all surveys. This differs a little from the gender distribution in higher education in Norway (around 60% women). The age range is similar across studies (18-35) and the mean age was 23.1 in 2018, 24,1 in 2021 and 24,0 in 2022. About half of the participants in all three samples reported being single. Ethnicity across the three SHoT samples was also relatively stable, with 8-10% percent being immigrants, defined as either the student or their parents being born outside Norway.

**Table 1:** Sociodemographic and Clinical Characteristics of the Three SHoT Studies

Characteristics	Men		Women		Total	
SHoT 2018						
Age, mean (*SD*)	23.4	(3.0)	23.0	(3.0)	23.1	(3.0)
Gender, % (*n*)	30.9%	(15,399)	69.1%	(34,437)		
Single, % (*n*)	56.2%	(8617)	47.5%	(16,238)	49.9%	(24,855)
Ethnicity, % (*n*)						
Norwegian	91.8%	(14,137)	92.1%	(31,711)	92.0%	(45,848)
Non-Norwegian	8.2%	(1262)	7.9%	(2726)	8.0%	(3988)
T-ILS score, *M* (*SD*)	7.13	(3.06)	7.66	(3.05)	7.50	(3.06)
SHoT 2021						
Age, *M* (*SD*)	24.3	(5.0)	24.1	(5.2)	24.1	(5.2)
Gender, % (*n*)	34.2%	(21,405)	65.6%	(40,807)		
Single, % (*n*)	55.1%	(11,777)	48.5%	(19,756)	50,8%	(31,533)
Ethnicity, % (*n*)						
Norwegian	91,3%	(19,542)	91.4%	(37,305)	91.4%	(56,847)
Non-Norwegian	8.7%	(1,863)	8.6%	(3,502)	8.6%	(5,365)
COVID-19 positive	3.1%	(622)	2.8%	(1091)	2.9%	(1703)
T-ILS score, *M* (*SD*)	8.64	2.98)	9.41	(2.87)	9.15	(2.93)
SHoT 2022						
Age, *M* (*SD*)	24.3	(3.3)	23.8	(3.2)	24.0	(3.2)
Gender, % (*n*)	33.6%	(17,939)	66.4%	(35,423)		
Single, % (*n*)	44.7%	(8023)	51.2%	(18,142)	49.0%	(26,165)
Ethnicity, % (*n*)						
Norwegian	89.6%	(16,080)	89.6%	(31,741)	89.6%	(47,821)
Non-Norwegian	10.4%	(1859)	10.4%	(3682)	10.4%	(5541)
COVID-19 positive	48.6%	(9636)	47.9%	(18905)	48.1%	(28,541)
T-ILS score, *M* (*SD*)	7.60	3.05	8.23	2.95	8.02	3.00

At the time of the SHoT 2021 data collection, 2.4% of the sample had tested positive for COVID-19, while 48.1% reported having tested positive by the time of SHoT 2022.

### Changes in Loneliness From 2018 to 2022

**Figure 1:**
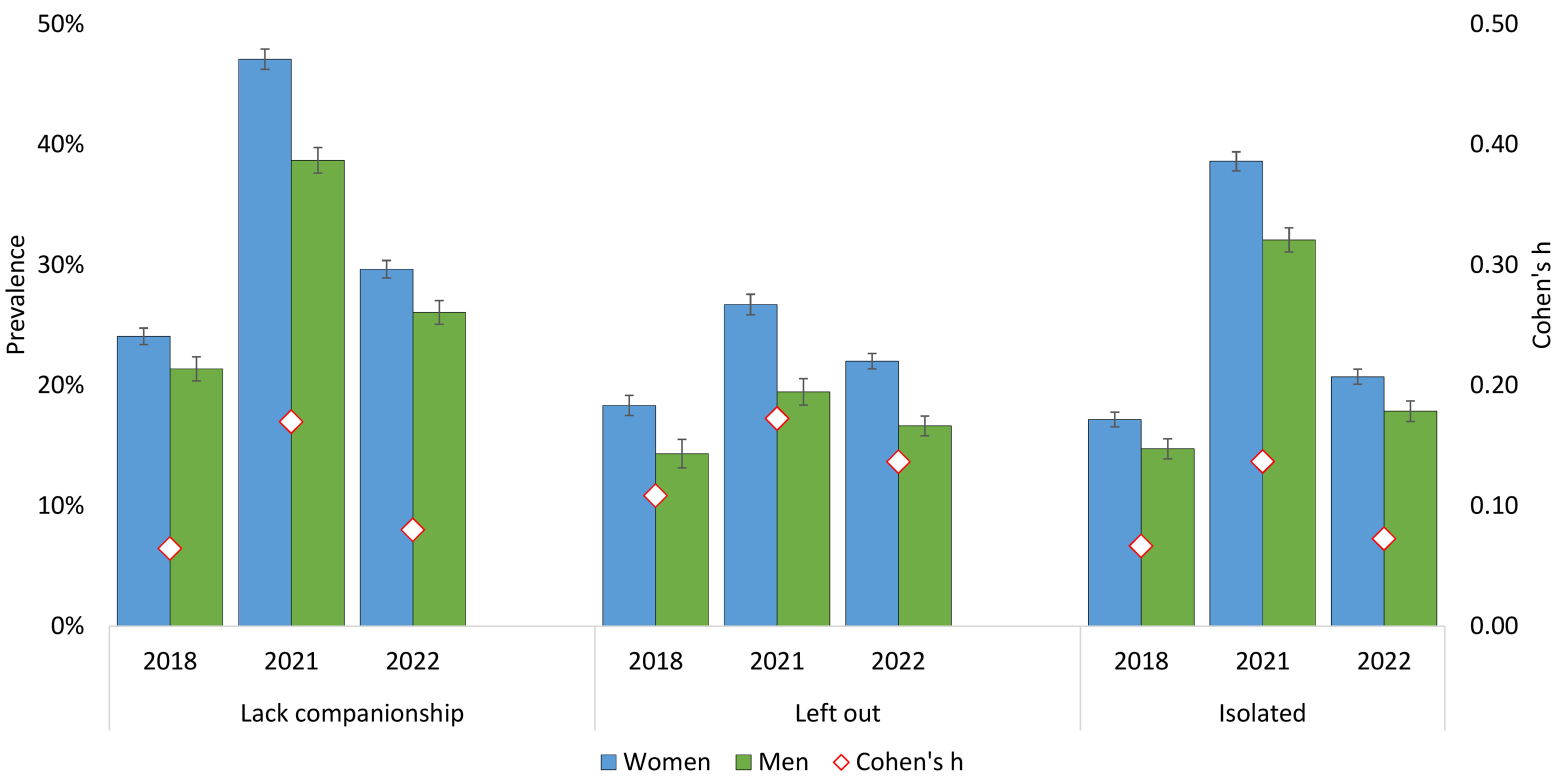
Trend in Loneliness From 2018 to 2022 Among Female and Male Students in the SHoT Study *Note.* Red diamonds represent gender differences expressed as Cohen’s *h*.

There was a sharp increase in loneliness across all three T-ILS items from 2018 to 2021 (see Figure 1 for details). And while the prevalence of loneliness decreased from 2021 to 2022, the levels of loneliness were still higher in 2022 than before the pandemic in 2018. For example, 47.1% of female students reported “often” or “very often” lacking companionship during the pandemic in 2021, while the corresponding estimates before the pandemic (2018) and after pandemic restrictions were lifted (2022) was 24.1% and 29.6%, respectively. This trend was similar for male students too, but as detailed in Figure 1 (red diamonds indicating Cohen’s *h*), the gender differences showed that females reported more loneliness in 2021, compared to both 2018 and 2022.

### Age Differences in Loneliness

**Figure 2:**
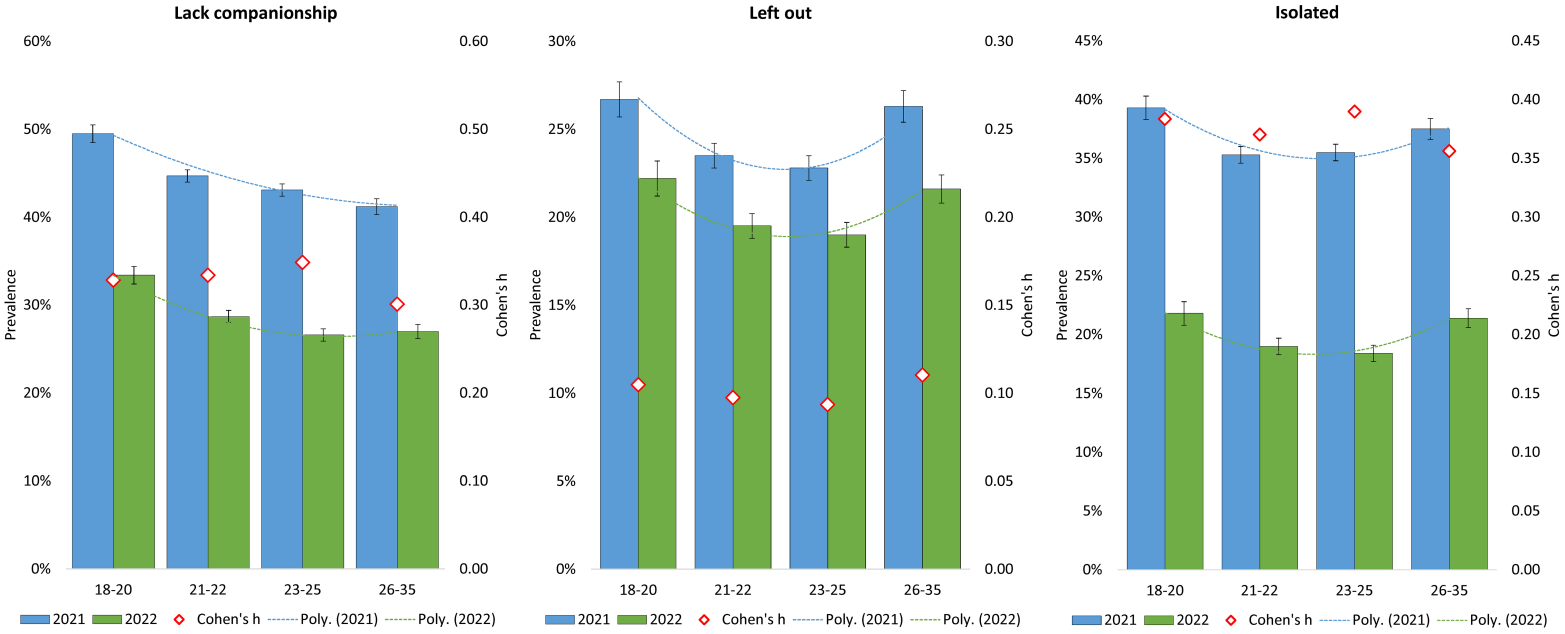
Loneliness and Age Group (in Men and Women Combined) in the SHoT 2021 and SHoT 2022 Studies *Note.* Red diamonds represent differences between 2021 and 2022 expressed as Cohen’s *h* (with 95% confidence intervals).

Figure 2 shows the prevalence of the three loneliness items across the different age groups in the SHoT 2021 and SHoT 2022 studies. As indicated by the dotted trend lines, there was a significant curvilinear relationship (all *p*s < .001) on feeling left out and isolated; both the youngest and oldest age-groups reported higher levels of feeling left out and feeling isolated (see Figure 2 for details). For the item on lacking companionship, the trend was more linear; the younger the student – the more they lacked companionship. The magnitude of differences between 2021 and 2022 was largest for feeling isolated and lacking companionship, with Cohen’s *h* effect sizes of around 0.4 and 0.3, respectively.

### Geographical Differences in 2021

**Figure 3:**
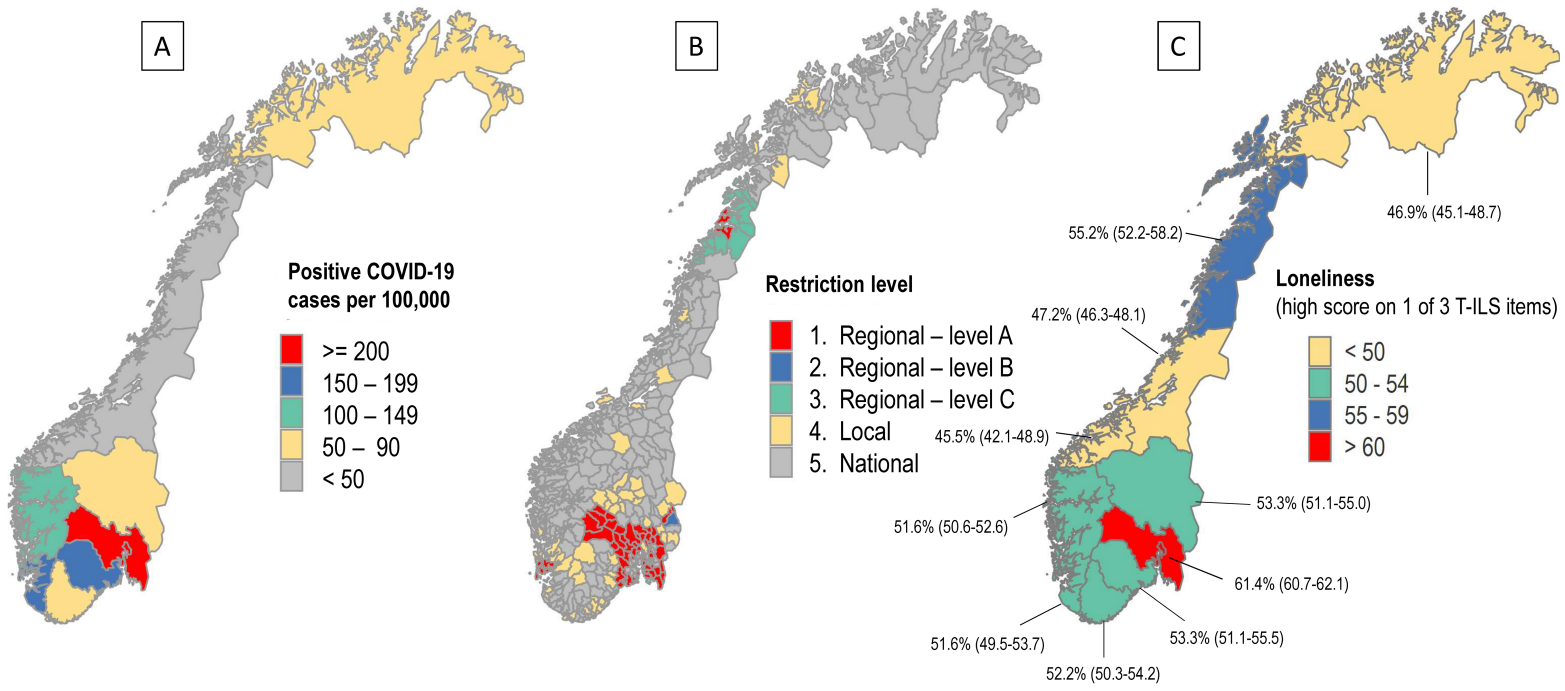
Geographical Differences in Number of Positive COVID-19 Cases (Panel A), COVID-Related Restrictions (Panel B) and Loneliness Prevalence (With 95% Confidence Intervals) in the SHoT 2021 Study (T-ILS; Panel C) *Note.* Data for all three figures are based on the situation in March (only) 2021. Sources: A–B: The Norwegian Institute of Public Health. $ Estimated loneliness prevalence (any of the three T-ILS items “often” or “very often”), adjusting for sociodemographic and COVID-19 factors (# of tests, positive test, quarantine).

There were large geographical differences in COVID-19 cases, as displayed in Figure 3; Panel A. In March 2021, the South-Eastern region surrounding the capital of Oslo and parts of Northern Norway had substantially more COVID-19 cases compared with other areas in Norway. As displayed in Figure 3; Panel B, there were also large geographical variations in terms of imposed COVID-19-related restrictions in March 2021. As expected, the strictest measures (marked in red) followed the same geographical distribution as the COVID-19 cases. Although the SHoT waves in 2018 and 2022 found no geographical differences in loneliness (data not shown), the 2021 survey revealed significant geographical differences in adjusted levels of loneliness during the data collection in March 2021. As displayed in Figure 3; Panel C, students studying at an institution in the South-Eastern region (marked in red) and parts of Northern Norway (marked in orange), reported significantly more loneliness compared with other geographical regions, after adjusting for sociodemographic-related and COVID-19-related factors.

### Loneliness and Campus Closure in 2021

**Figure 4:**
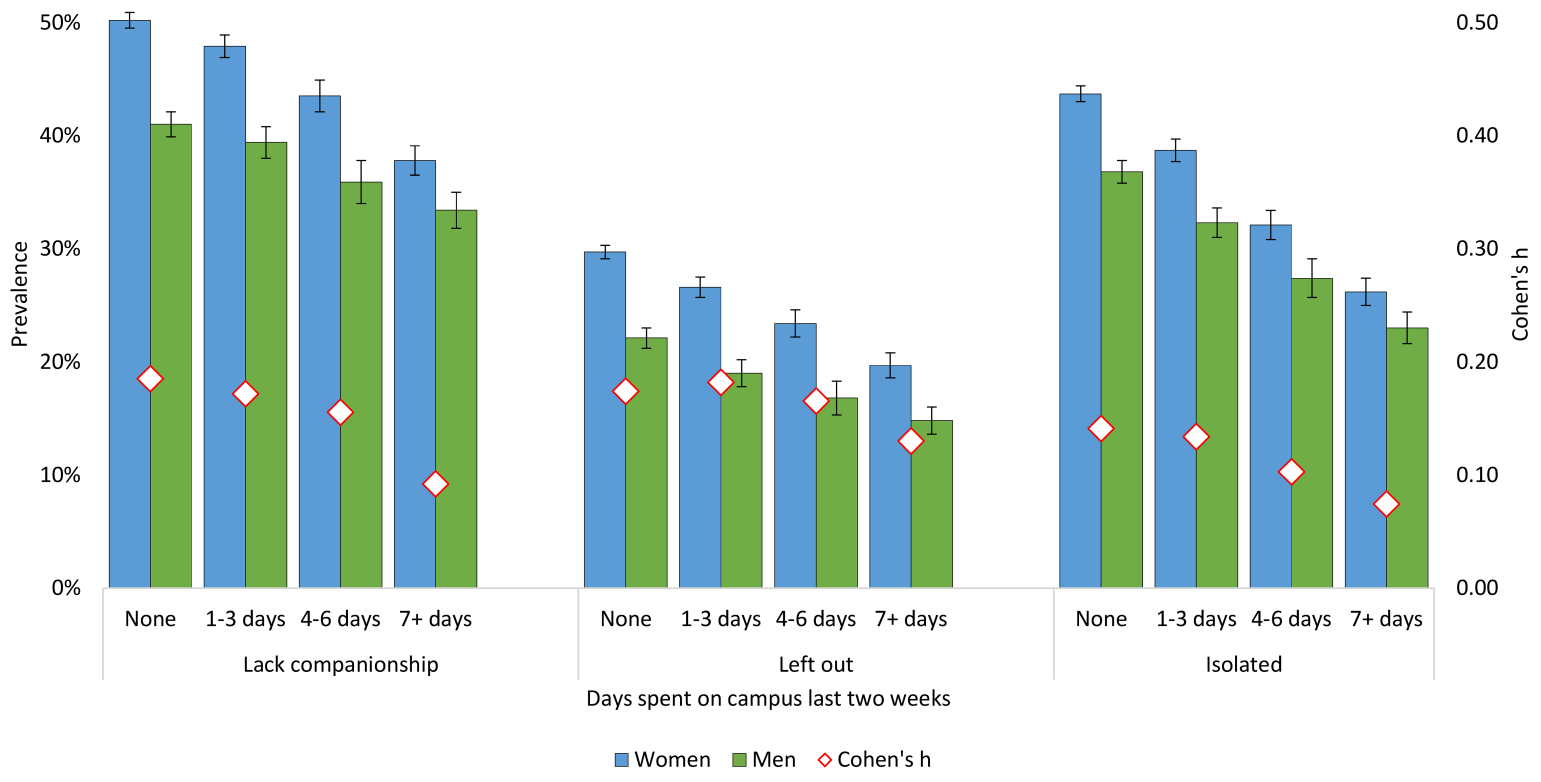
Loneliness by Campus Closure Due to COVID-19 in the SHoT 2021 Study *Note.* Red diamonds represent gender differences expressed as Cohen’s *h*.

Figure 4 displays the association between loneliness and campus closure in the SHoT 2021 study. There was a significant negative dose–response association between all three T-ILS items and days spent on campus. Students spending 7+ days on campus during the last 2 weeks, reported significantly less loneliness during this period, compared with students who were not permitted on campus, after adjusting for sociodemographic and COVID-19-related factors. The trend was similar for both male and female students.

### Loneliness and Remote Learning in 2022

**Figure 5:**
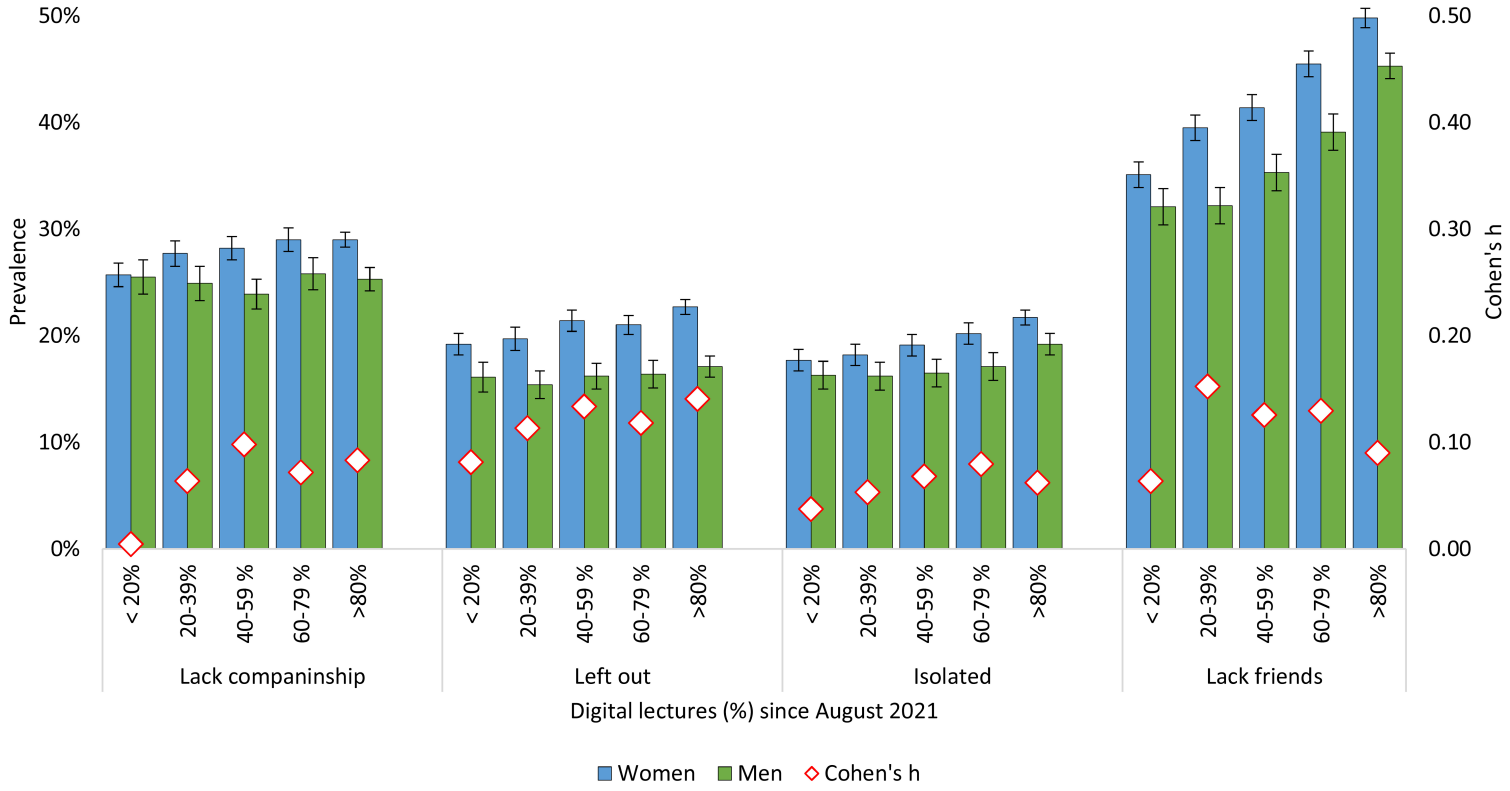
Loneliness and Lack of Friends by Degree of Digital Lectures in the SHoT 2022 Study *Note.* Red diamonds represent gender differences expressed as Cohen’s *h*.

As displayed in Figure 5, there was also a significant dose–response association between all loneliness items and the use of online lectures in 2022. Students who had their physical classes replaced by online lectures in 80-100% of the time since August 2021, reported significantly more loneliness compared to students who had more in person teaching. This graded association was present for all T-ILS items, but was especially strong for the item assessing to what extent students lacked friends at their place of study. For example, among female students who had predominantly remote learning, 49.8% reported having “no” or “few” friends, compared to 35.1% among those who had less than 20% of online lectures. The trend was similar for male students.

## Discussion

The study showed a significant increase in loneliness during 2021 compared to the 2018 pre-pandemic SHoT survey. The level of loneliness was highest in regions with high COVID-19 associated restrictions in 2021 and among those students who did more remote learning, as well as among the youngest students. The 2022 SHoT study showed loneliness reduced significantly from 2021 but was still higher relative to pre-pandemic levels. Females consistently showed higher levels of loneliness, relative to men, with these gender differences increasing during the pandemic. Prior research has suggested that higher levels of loneliness in females may be due to a greater sensitivity of females to interpersonal relationships starting at adolescence (Maes et al., 2019). Further, the increased rate of mental health problems for women during the pandemic was partly explained by loneliness, underscoring the adverse consequences of loneliness (Dotsikas et al., 2023)

Together, these results confirm the higher levels of loneliness during the COVID-19 pandemic experienced by young adults that have reported in previous studies (Horigian et al., 2021; Padmanabhanunni & Pretorius, 2021; Sigfridsson & Brandt, 2021). Similar levels have also been found in the general population during the pandemic (Ernst et al., 2022). The increase in loneliness, with a twofold increase in some items that comprise the loneliness measure, confirms young adults are at high-risk group for loneliness. The rise in off campus online lectures seems to have been particularly difficult for female students, who reported feeling more socially isolated and lonely than students who were less affected by campus restrictions.

The results confirm a trend of loneliness as an increasing public health concern (Lim et al., 2020), given that the level of loneliness has shown a gradual increase from 2010 until 2022, in addition to the time limited peak during the pandemic (Hysing et al., 2020). Still, a meta-analysis has shown lower rates of loneliness among northern European countries compared to other geographical regions, and thus this may indicate that loneliness rates are even higher in other countries (Surkalim et al., 2022). The high rate of loneliness is especially worrisome given it is an established risk factor for both mental and physical health problems in this age group (Christiansen et al., 2021). Consistent with this finding, the increase in depression symptoms observed in a study of young adults may be due to this the rise in loneliness (Horigian et al., 2021). Although beyond the scope of the present study, future studies should investigate how loneliness is associated with later mental and physical health in young adults.

The unprecedented high levels of loneliness among young adults in higher education seem to be driven largely by the restriction levels which impacted on the formation of normal friendship patterns. This is in line with previous studies which have found higher levels of loneliness among students during lockdown periods in comparison to times with less restrictions (Macalli et al., 2022) The regional differences in loneliness related to the impact may be accounted for by a range of restrictions, both restrictions directly related to being a student, such as campus lock downs and online teaching, but also on more general restrictions on social contact. Still, the dose-response associations between days on campus in 2021, and similarly to online teaching in 2022, raises the potential importance of live face-to-face instruction on student’s loneliness. This may be especially important to establish social relationships in the class and student group, which is indicated by the strong association between the proportion of online teaching and having friends at the study site.

The strength of the present study is that the surveys are similar in inclusion and recruitment across the three data collections and have identical measures of loneliness. The results should be interpreted in light of some limitations. The attrition rate is high across the health surveys with no information about non-participants other than age and gender. We cannot exclude the possibility of selective attrition among those with health problems. Also, it is possible that students who a particularly lonely may not participate. If so, the results might have underestimated the true level of loneliness in the population. The loneliness measure is a well-validated and commonly used assessment of loneliness, but it is an indirect measure and does not ask the participants directly if they fell lonely as has been done in some previous studies (Wickens et al., 2021) Further, the data collection has been done at set time points, and more frequent assessments could have given more detailed information about stability and changes in loneliness across the pandemic and restriction levels. Regarding the reported rates of COVID-19, these are uncertain and could be an underestimation due to the lack of testing and confirmation of COVID-19.

The current results confirm the adverse public health consequences of the COVID-19 pandemic and related pandemic restrictions. In Norway, higher education was one of the domains with high levels of restrictions (Helsingen et al., 2020). When governments and health officials are making decisions regarding restrictions, the results underscore the need to consider the adverse psychological consequences of restrictions in addition to direct health impact. The study helps to identify high risk groups and predictors of loneliness that could inform policy and interventions to reduce harm in these groups.

Further, the results of the present study confirm loneliness as a major public health concern among young adults in higher education and interventions in these settings may be needed. The youngest students were at higher risk, and this indicates the importance of supporting young adults in establishing a social network during the transition to university- and college life. There are available and effective interventions to reduce loneliness, however, they have mainly been tested in high risk groups and with individual or group based approaches (Eccles & Qualter, 2021). There are still relatively few interventions to reduce loneliness among young adults (Hawkley et al., 2022). Identifying predictors of loneliness among young adults may also give insights into how we can reduce loneliness by systemic changes. For instance, finding the right balance between online teaching and physical presence for students may be areas that need to be considered both in response to future pandemic restrictions and when planning for teaching in higher education post pandemic. At present, higher learning institutions are redesigning their teaching to find the balance between in-person and digital presence, and preventing loneliness and establishing social relationships is an important aspect to consider.
